# Antiobesity and Antioxidant Potentials of Selected Palestinian Medicinal Plants

**DOI:** 10.1155/2018/8426752

**Published:** 2018-06-13

**Authors:** Rana M. Jamous, Salam Y. Abu-Zaitoun, Rola J. Akkawi, Mohammed S. Ali-Shtayeh

**Affiliations:** Biodiversity and Environmental Research Center (BERC), Til, Nablus, State of Palestine

## Abstract

We evaluated the antioxidant and porcine pancreatic lipase inhibition (PPLI) activities of 90 plants extracts. The antioxidant activity was measured using the free-radical scavenging capacity (DPPH) and reducing power (RP) assays. The pancreatic lipase inhibition assay was used to determine the PPLI activity of plant extracts. Among the 90 plant extracts examined, 41.0 % crude extracts showed antilipase activity of more than 50%. The most active plants by means of IC_50_ value were* Camellia sinensis* (0.5 mg/ml),* Ceratonia siliqua *(leaves) (0.8 mg/mL),* Curcuma longa *(0.8 mg/mL),* Sarcopoterium spinosum* (1.2 mg/mL), and* Mentha spicata* (1.2 mg/mL). The antioxidant activity of plant extracts using the DPPH and RP assays reveals comparable results. The most active antioxidant extracts using both assays were the leaves and fruit epicarp of* Rhus coriaria*, areal parts of* Sarcopoterium spinosum*, and leaves of* Ceratonia siliqua*. Our results suggest natural resources that possess strong antioxidant and pancreatic lipase inhibitory activities with potential applications in the treatment and prevention of obesity and overweight. The extracts of* Camellia sinensis, Ceratonia siliqua, Curcuma longa, Sarcopoterium spinosum, *and* Mentha spicata* were proved to have a great potential as antioxidants and antiobesity agents.

## 1. Introduction

Overweight and obesity are defined as abnormal or excessive fat accumulation that may impair health. Body mass index (BMI) is a crude population measure of obesity that is commonly used to classify overweight and obesity in adults; it is defined as a person's weight in kilograms divided by the square of their height in meters (kg/m2). A person with a BMI of 30 or more is generally considered obese, while a person with a BMI equal to or more than 25 is considered overweight [1].

The prevalence of obesity differs from one country to another and depends on several factors including gender, age, educational accomplishment, annual household income, employment status, and social class [2, 3]. Obesity is considered an extremely costly health problem, the direct medical cost of overweight and obesity combined is approximately 5.0% to 10% of US health care spending [4].

According to the recent WHO report, more than 1.9 billion adults, 18 years and older, were overweight in 2016, of these over 650 million were obese. Over 340 million children and adolescents aged 5-19 were overweight or obese, while 41 million children under the age of 5 were overweight or obese in 2016. Most of the world's population lives in countries where overweight and obesity kill more people than underweight; obesity has reached epidemic proportions globally, with about 2.8 million people dying each year as a result of being overweight or obese [1].

Overweight and obesity were once considered a high-income countries problem; however, it is now also prevalent in low- and middle-income countries [1]. The factors leading to this widespread increase in obesity have been suggested to include economic growth, modernization, westernization of lifestyles (high calorie diet including processed foods higher in fats and refined sugars and decrease in exercise levels), and the globalization of food markets [5, 6]. Other factors may also contribute to this problem including familial susceptibility, endocrine disorders, and environmental factors [7], with women being suggested to be especially at risk [8, 9].

In Palestine, the prevalence of overweight and obesity was estimated as 58.7% and 71.3% among men and women, respectively [10]. This can be attributed to several factors including decreased physical activity, increased consumption of high caloric foods, particularly with an increase in energy coming from fat, smoking, and urbanization [11]. A cross-sectional study was carried out in Palestine to investigate the prevalence of overweight and obesity among school children aged from 6-12 years. The study revealed that the prevalence of overweight and obesity among male students was 13.3% and 7.9%, respectively, while it was 13.6% and 4.9% among female students, respectively [12].

Different powerful synthetic chemical drugs used against obesity are available in the pharmaceutical market; the FDA has approved five prescription medications for long-term use for the treatment of overweight and obesity, including orlistat, lorcaserin, phentermine-topiramate, naltrexone-bupropion, and liraglutide [13]. However, the cost of these products generally keeps them out of reach of most people; more importantly, many of these products have adverse side effects including cardiovascular events and strokes, coughing, dizziness, mouth dryness, anxiousness, fatigue, flatulence, headache, insomnia, leakage of oily stools, nausea, and hepatic adverse effects [14–17]. That is the reason for the withdrawal from the market of some older drugs such as Sibutral, rimonabant, isomeride, ponderal, and Xenical [16, 18]. Hence, the use of naturally occurring inhibitors is considered to be safer [19].

Pancreatic lipase plays a key role in the efficient digestion of triglycerides [20]. Lipases are involved in the hydrolysis of glycerides to glycerol and free fatty acids. The enzyme inhibition is one of the approaches used to treat obesity due to the fact that 50-70 % of total dietary fat hydrolysis was performed by pancreatic lipase [21].The mechanism involves inhibition of dietary triglyceride absorption, as this is the main source of excess calories [22]. Besides, pancreatic lipase inhibition does not alter any central mechanism which makes it an ideal approach for obesity treatment [23]. This enzyme has been widely used for the determination of the potential efficacy of natural products as antiobesity agents [15]. Orlistat is the synthetic clinically approved drug used for obesity management. This molecule acts by the inhibition of PL activity and reduction of triglyceride absorption, and its long-term administration accompanying an energy restricted diet results in weight loss [24].

The impact of overweight and obesity from a public health perspective is enormous and continue to increase. Numerous studies have verified the association of overweight and obesity in the development of different metabolic disorders including diabetes mellitus, atherosclerosis, cardiovascular diseases, hypertension, and cancer [25–27]. It is therefore essential to develop ways of preventing more people from becoming obese by finding natural inhibitors of digestion and absorption of dietary lipids which reduce energy intake through gastrointestinal mechanisms without altering any main mechanisms [22].

Plant derived products (fruits and vegetables) constitute an important part of the human diet all over the world and provide nutrients that are essential for life. Plants contain nonnutrient biologically active phytochemicals including polyphenols and anthocyanins, which are involved in a wide range of health benefits such as decreasing the rates of cardiovascular diseases, lowering the risk of cancer, stroke, and type 2 diabetes, and preventing obesity [28, 29]. Antioxidant-rich fruits help in the prevention of free-radical induced oxidative stress [30–32].

Plant derived products may also contain phytochemicals that act as enzyme inhibitors [33]. These compounds have the ability to bind to enzymes and inhibit their activity. The presence of inhibitors of carbohydrate and glycerides hydrolyzing enzymes such as *α*-glucosidase, *α*-amylase, and lipase in plant derived products helps in the control of blood glucose level in patients with type 2 diabetes and to prevent obesity [20, 34].

Knowledge of herbs has been inherited and transferred from generation to generation for thousands of years. In Palestine as in many other countries herbal medicine is still the primary form of therapy in the practiced traditional medical systems [35–39]. Three hundred ninety-six plant species have been reported to be used in Traditional Arabic Palestinian Herbal Medicine (TAPHM) for the treatment of various diseases [40]; of these 39 plants were reported to be used for the treatment of obesity [41] (Table 1). Accordingly, the aim of this study was to evaluate in vitro the antioxidant and PPL inhibitory activities of some Palestinian medicinal plants that have been reported in TAPHM for the treatment of obesity and other chronic diseases and to explore newly potent and safe natural therapeutic agents for the treatment of obesity.

## 2. Materials and Methods

### 2.1. Plant Materials

Plants material were either collected from Nablus area in Northern Palestine or purchased from traditional drug stores (Attarin Shops) in Nablus city. Taxonomic identification of the plant materials was kindly confirmed by Professor M. S. Ali-Shtayeh, plant biologist, BERC. The voucher specimens have been deposited at the Herbarium of the Biodiversity & Biotechnology Research Institute, BERC, Til, Nablus, Palestine.

Seventy-eight plant species used in TAPHM, of which 39 species were reported to be used for the treatment of obesity (Table 1) [41], have been analyzed in this study for their antioxidant and pancreatic lipase inhibition potentials. Plant parts were dried in the shade, ten grams from each dried material was ground and incubated separately with 100 ml of 70% ethanol at 35°C for 3 hours. The extracts were then filtered and evaporated under vacuum at 50°C to dryness. The powdered extracts were stored at −20°C for further analyses.

### 2.2. Pancreatic Lipase Inhibition Assay

#### 2.2.1. Lipase Preparation

The enzyme solution was prepared immediately before use following the method described by Bustanji et al. [42] with some modifications. Crude porcine pancreatic lipase type II (Sigma, USA, EC 3.1.1.3) was suspended in 20 mM tris-HCl buffer pH 8 to give a concentration of 10 mg/ml. The suspension was mixed using a stirrer for 15 min and centrifuged at 1500 g for 10 min and the clear supernatant was recovered.

#### 2.2.2. Lipase Inhibition Reaction

The ability of the plant extracts to inhibit PPL was measured using the modified method previously reported by Bustanji et al. [42]. The lipase activity was determined by measuring the hydrolysis of p-nitrophenol butyrate (pNPB) to p-nitrophenol at 410 nm using UV-VIS spectrophotometer. Lipase assays were performed by incubating 200 *μ*l of plant extract (5mg/ml ethanol) with 100 *μ*l of PPL solution for 5 min at 37°C; then 10*μ*L of the pNPB substrate (100 mM in acetonitrile) was added. The volume was completed to 1 mL using the buffer. The release of pNPB is estimated as the increment increase in absorbance against blank. The percentage of residual activity of PL was determined by comparing the lipase activity in the presence and the absence of the tested inhibitors. Orlistat (200 *μ*g/ml) was used as a positive standard inhibitor control, whereas plant extract was replaced by ethanol to be used as negative control. All experiments were repeated twice.

Inhibition of the lipase activity was expressed as the percentage decrease in the activity when PPL was incubated with the test compounds. Lipase inhibition (%) was calculated according to the following formula:

(1) Lipase  I%=100×Δ  A  Control−Δ  A  SampleΔ  A  ControlΔ  A  Control=A  Control−A  BlankΔ  A  Sample=A  Test−A  Blank

#### 2.2.3. Minimum Inhibitory Concentration* IC*_50_

The concentration of most active plant extracts giving 50% lipase inhibition (IC_50_) was performed using several concentrations of the extracts, ranging from 0.156 to 5.0 mg/mL and the percentages of residual activity of PL data were used to evaluate the IC_50_ values. The IC_50_ value of the extract was calculated from the least squares regression line of the semilogarithmic plot against percentage inhibition curve using Microsoft Excel version 10 software. All assays were repeated twice on different occasions, and the calculated inhibition percentages were the mean of 2 observations.

### 2.3. Antioxidant Capacity Assays

#### 2.3.1. DPPH (*α*, *α*-Diphenyl-*β*-picrylhydrazyl) Assay for Scavenging Activity

DPPH radical scavenging activity was determined according to the method described by Choi et al. [43]. Briefly, 1 ml of the ethanolic plants extract (1mg/ml) or standards (BHT and ascorbic acid) were mixed with 1.5 ml (0.02 %) of DPPH solution in methanol. The mixture was incubated in the dark for 30 min, and the absorbance was measured at 517 nm in the spectrophotometer (6800 UV-VIS spectrophotometer) using methanol as blank. The percentage of scavenging of DPPH radicals was calculated by using the following formula [27]:

(2) $$\begin{array}{c} Scavenging\;\;Percentage\left(\;\%\;\right)=100-\left[\;\left(\;\frac{AS}{AC}\;\right)x100\;\right] \end{array}$$

where* AS* is the absorbance of the sample;* AC* is the absorbance of the negative control (methanol without the sample).

The IC_50_ value, which is defined as the concentration of extract (mg mL-1) required to scavenge 50 % of DPPH, was calculated using the graph by plotting inhibition percentage against serial extract concentration (1.0-0.016 mg/ml) [44]. The antioxidant activity index (AAI) was calculated by dividing the DPPH final concentration (0.1 mg/ml) by the IC_50_ of the plant extract [45].

#### 2.3.2. Reducing Power Capacity Assessment

The reducing power (RP) was evaluated following the method of Oyaizu [46]. In this technique, 1.0 ml of various concentrations of plants extracts (1.0-0.016 mg/ml) were mixed with 2.5 ml of potassium buffer (0.2 M, pH 6.6) and 2.5 ml of potassium ferricyanide [K_3_Fe (CN) 6] (1 %) solution. The mixture was incubated for 30 min at 50°C, and then 2.5 ml of trichloro acetic acid (10 %) solution was added to the mix. The total mixture was centrifuged at 3000 g for 10 min. Then 2.5 ml supernatant solution was withdrawn from the mixture and mixed with 2.5 ml of distilled water and 0.5 ml of FeCl3 (0.1 %) solution. Blank sample was similarly prepared by replacing extract with 60% ethanol, to calibrate the instrument (6800 UV-VIS spectrophotometer). The absorbance of the solution was measured at 700 nm; higher absorbance indicates higher RP. BHT and ascorbic acid were used as standards.

The effective concentration at which the absorbance was 0.5 for reducing power (EC50) was obtained by interpolation from linear regression analysis [47]. Increased absorbance of the mixture reaction indicates increasing RP [48].

### 2.4. Statistical Analysis

All results were expressed as mean ± standard deviation (n=3). Significance of differences from the control was determined by Duncan's test and a* p value < 0.05* was considered significant.

## 3. Results

Table 2 summarizes the results of antioxidant and antilipase inhibitory assays using ethanolic extracts from 78 medicinal plants (90 plants extracts) commonly administrated orally or topically to treat diverse diseases.

Using the pancreatic lipase inhibition assay, the pancreatic lipase inhibition activity was determined by measuring the hydrolysis of pNPB to p-nitrophenol at 410 nm [42]. The antioxidant activity was measured using the free-radical scavenging capacity (DPPH) and RP assays. The RP was evaluated by measuring absorbance at 700 nm after mixing the samples with ferric compounds; higher absorbance indicates higher RP. The scavenging effects on DPPH radicals were determined by measuring the decay in absorbance at 517 nm due to the DPPH radical reduction, indicating the antioxidant activity of the compounds in a short time [49, 50].

Pancreatic lipase inhibition was expressed in percentage (%) and IC_50_, whereas antioxidant activity was expressed as AAI and EC50 using DPPH and RP capacity assessment techniques, respectively.

**Figure 1:**
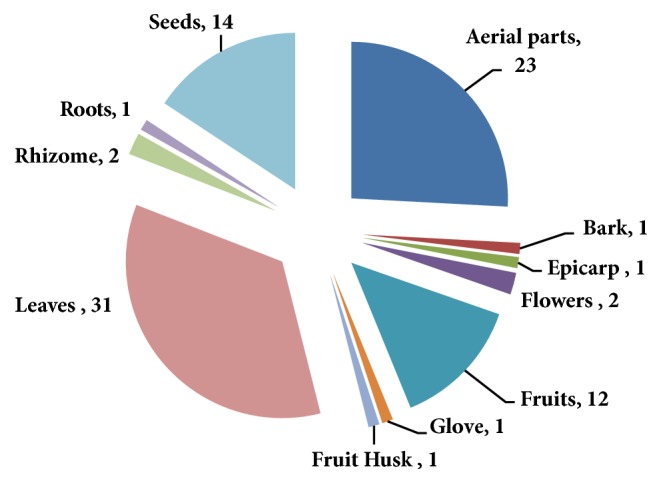
Plant parts used in the study.

Of the 90 ethanolic crude extracts, 31 were prepared from leaves, 23 from aerial parts, 14 from seeds, 12 from fruits, two from each rhizomes, fruit husk, and flowers, and one from the epicarp, root, gloves, and bark (Figure 1). The selection of plants part was primarily dependent on the significant role in traditional medication or cooking purposes [41, 51].

### 3.1. Antioxidant Activity

**Figure 2:**
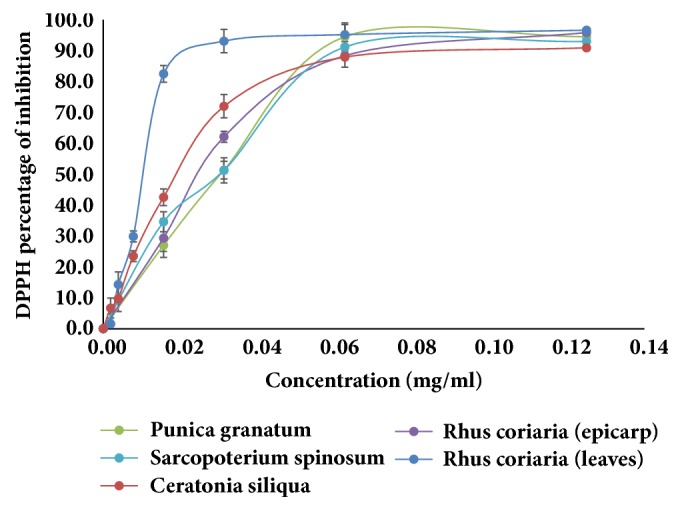
Antioxidant activity of the five most active plants' ethanolic extract using DPPH. Experiments have been performed in triplicate.

Antioxidant activity of plant extracts using the DPPH assay ranged between 1.0 and 95.1% for plant extracts at a concentration of 1 mg/ml. Sixty-one extracts exhibited antioxidant activity ≥ 50 %. The IC_50_ values for the leaves and epicarp of* Rhus coriaria*, aerial parts of* Sarcopoterium spinosum*, leaves of* Ceratonia siliqua*, and the fruit husk of* Punica granatum* were 0.02, 0.02, 0.02, 0.04, and 0.05 mg/ml (Figure 2), respectively. Twenty-one plant extracts exhibited AAI > 0.5; the antioxidant activity indices (AAIs) for* Rhus coriaria* leaves and epicarp,* Sarcopoterium spinosum*, and* Ceratonia siliqua* were higher or comparable to the AAI of ascorbic acid (3.85) (Table 2). This indicates the high potential for free-radical scavenging of these extracts.

The RP was measured by direct electron donation in the reduction of Fe^3+^(CN^−^)^6^–Fe^2+^(CN^−^)^6^. The product was visualized by forming the intense Prussian blue color complex and then the absorbance was measured at the 700 nm; a higher absorbance value indicates a stronger RP of the samples.

**Figure 3:**
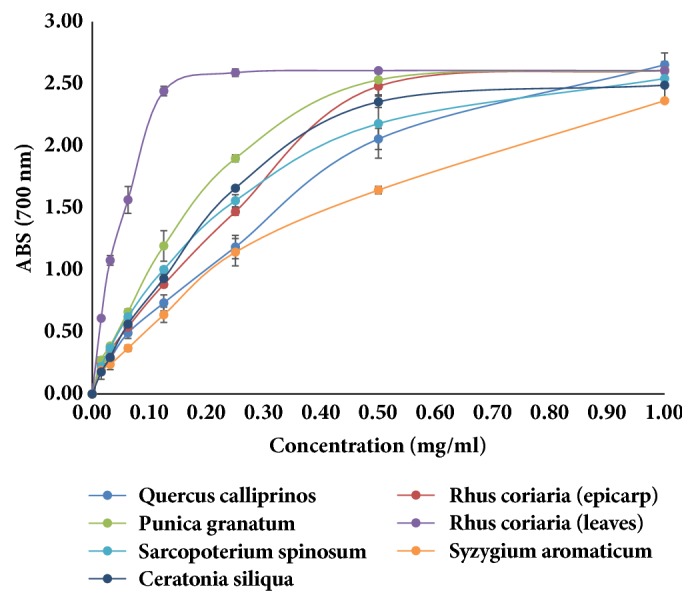
Antioxidant activity of the seven most active plants extracts using reductive potential. Experiments have been performed in triplicate.

Using ferric reducing power assay, the EC_50_ value for the ethanolic extracts ranged between 1.6 and 0.005 mg/ml. The EC_50_ of the leaves of* Rhus coriaria*, fruit husk of* Punica granatum*, areal parts of* Sarcopoterium spinosum*, leaves of* Quercus calliprinos*, leaves of* Ceratonia siliqua*, and epicarp of* Rhus coriaria *were 0.005, 0.03, 0.04, 0.05, and 0.06 mg/ml, respectively, whereas the EC_50_ value for the positive controls ascorbic acid and BHT was 0.057 mg/ml for both controls (Figure 3, Table 2). These results indicate the relatively high ferric reducing activity of these medicinal plant extracts compared to the pure standards ascorbic acid and BHT.

### 3.2. Pancreatic Lipase Enzyme Inhibition Activity

Ninety crude extracts were prepared from natural plant species, either collected from the Nablus area or purchased from traditional drug shops, and their antilipase activity was investigated at a concentration of 5 mg/mL for PPL inhibition. A variety of the tested plant extracts shows a strong inhibitory potential to the pancreatic lipase digestive enzyme (Table 2); the PPL inhibitory activity of the tested plant extracts ranged between 1.7 and 98.97 %. The plant extracts were divided into three categories, which were low (< 30%) inhibition, medium (30-70%) inhibition, and high (> 70%) inhibition when incubated with PPL for 10 min at 37°C. The PPL inhibition assay screening detected 29 extracts exhibiting high (> 70%) inhibition while 23 extracts with medium (30-70%) inhibition and the remaining 38 plant extracts showed low inhibition (< 30%). All plants extracts were set at 5 mg/ml as this concentration could give a consistent result with low standard deviation in the assay.

The significant inhibition of PPL was observed up to 99.0 % with* Camellia sinensis* leaves, 97.4% with* Curcuma longa* rhizomes, and 96.8 of* Elaeagnus angustifolia* leaves. Treatment with orlistat (at final concentration 200 *μ*g/mL) as a positive control, a well-known antilipase agent, significantly inhibited the PPL activity up to 92.0 %. Orlistat, a hydrogenated derivative of lipstatin, is the only pancreatic lipase inhibitor currently approved for a long-term treatment of obesity.

**Figure 4:**
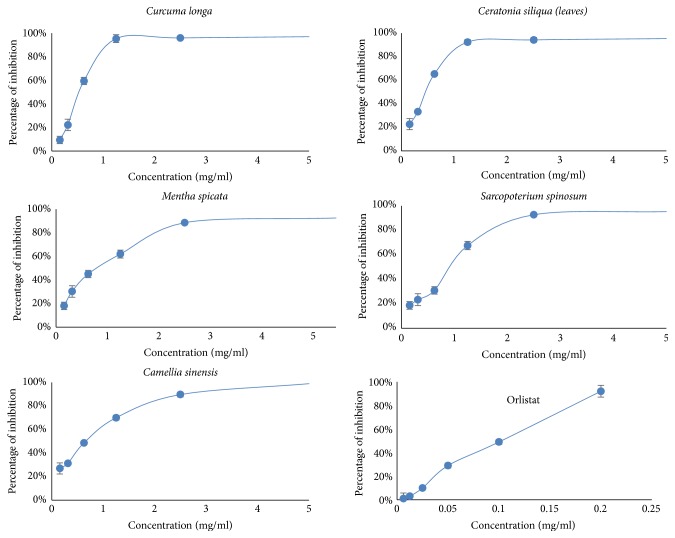
Porcine pancreatic lipase inhibitory (PPLI) activity of* Camellia sinensis, Ceratonia siliqua*,* Curcuma longa, Sarcopoterium spinosum, *and* Mentha spicata. *Orlistat was used as a positive control. Experiments have been performed in triplicate.

Among the 90 plant extracts examined, 37 (41 %) crude extracts from natural plant species showed antilipase activity of more than 50%. These were further investigated for their PPL inhibitory effects at different concentrations, and a dose-response curve was obtained.  Figure 4 presents the top 5 plant extracts in comparison with orlistat as a control. The most active plants by means of IC_50_ value were* Camellia sinensis* (0.45 mg/ml),* Ceratonia siliqua *(leaves) (0.76 mg/mL),* Curcuma longa *(0.82 mg/mL),* Sarcopoterium spinosum* (1.17 mg/mL), and* Mentha spicata* (1.19 mg/mL).

Of the 41 plant extracts used by Palestinian in TAPHM for the treatment of obesity 16 plant extracts (39%) have shown possessing a strong PPL inhibitory activity > 70%, 7 with medium PPL inhibitory activity, while the rest (18) exhibited weak activity < 30 % (Table 2).

## 4. Discussion

With more people avoiding chemical drugs for the management of overweight and obesity, due to the fear of health adverse side effects, tendency is now towards natural-based products; thus the development of new antiobesity molecules from natural products has become a necessity. This seems doable because, in traditional herbal medicine, several plants are used for their weight- reducing effects. The plant bioactive constituents are expected to act as natural inhibitors of digestive lipases [22, 52]. Antioxidant and* in vitro *porcine pancreatic lipase, PPL, inhibitory tests were conducted on seventy-six plants, of which thirty-nine species with weight-reducing or related potential were used in Palestinian traditional medicine, to find new crude antiobesity products from natural sources.

In this study 59.3 % and 68.6 % of the plants extracts have shown possessing antioxidant activity using the RP and DPPH assays, respectively. The percentages of DPPH free-radical scavenging activity and ferric reducing power were found to be maximum at 1 mg/mL of the plant extract. The antioxidant activity of the plant extracts has shown being comparable using the DPPH and the RP assays (Table 2); both assays revealed similar antioxidant activity of the tested extracts except for a very small number of extracts (5 extracts, 5.5 %). The leaves and epicarp of* Rhus coriaria* exhibited the highest antioxidant activity in both assays. However, only 5 plant extracts,* Rubus sanctus*,* Coriandrum sativum, Zingiber officinale, Ruta chalepensis*, and* Retama raetam*, have shown possessing strong scavenging capacity by DPPH, while they exhibited low RP activity. Both DPPH and RP are known to be related to the nature of polyphenolics contributing to their electron transfer/hydrogen donating ability [53]. Thus, it is preferred to assess the antioxidant capacity of a plant extract by more than one method, as different methods can assess scavenging capacity of different free radicals and this can explain the difference in the antioxidant activity of the tested plant extracts using DPPH and RP assays. Free radicals are involved in many disorders like cancer, diabetes mellitus, and neurodegenerative and inflammatory diseases. Antioxidants due to their scavenging activity are useful for the management of those diseases [54, 55].

All plants analyzed in this study have been reported in TAPHM to be used for the treatment of one or more ailments including chronic diseases [40]. However, 39 of these plants which possess high antioxidant activity (> 50%) by either method, DPPH or RP, have been reported by the Palestinian people to be used as food, either tea (53.8 %), raw (43.6%), or seasoning (41.0 %). This would indicate the safety of using these plants as natural antioxidant agents for therapeutical uses.

Five basic mechanisms have been suggested for the weight-reducing effects of antiobesity natural products including inhibiting pancreatic lipase activity [56], promoting lipolysis [57], preventing adipogenesis [58], promoting thermogenesis and lipid metabolism [58], and controlling appetite [59]. However, pancreatic lipase inhibitory effect is one of the most widely studied mechanisms to determine the potential activity of natural products as obesity modulating agents [60].

Several studies have been conducted to evaluate the pancreatic lipase inhibitory activity of plant extracts; these studies have led to the identification of several plants and the isolation of natural compounds with PPL inhibitory activity [15, 27]. In our study, the most active PPLI plant extracts by means of minimum inhibitory concentration (IC_50_) were* Camellia sinensis, Ceratonia siliqua, Curcuma longa, Sarcopoterium spinosum, Mentha spicata*,* Rhus coriaria* L. (Anacardiaceae),* Dittrichia viscosa* (L.) Greuter (Asteraceae),* Rosmarinus officinalis* L. (Lamiaceae),* Eruca sativa* Miller (Brassicaceae), and* Elaeagnus angustifolia* L. (Elaeagnaceae) (Table 2).

In the following, we discuss some of these plants and their possible action mechanisms.

*Camellia sinensis* (tea) was among the most active plant extracts as PPLI in this study by means of IC_50_. Our results are in accordance with those of other researches which demonstrated that a tea-based drug (Exolise) standardized with the active component catechin at 25% was proposed as a natural antiobesity agent, showing strong PPLI activity and increase in thermogenesis [61].

A randomized, double-blind, placebo-controlled clinical trial was conducted to explore the effect of the consumption of high-dose of green tea extract on weight reduction [62]. The study revealed a significant weight loss, reduced waist circumference, and a consistent decrease in total cholesterol and LDL plasma levels without any side or adverse effects in women with central obesity [62]. Different types of tea are among the most widely investigated natural materials for pancreatic lipase inhibition. Various polyphenols (e.g., L-epicatechin, epicatechin gallate (ECG), epigallocatechin (EGC), and epigallocatechin gallate, EGCG) isolated from tea leaves showed strong inhibitory activity against pancreatic lipase [63]. The efficacy of tea polyphenols (e.g., catechins: epigallocatechin gallate, EGCG) in regulating multiple targets concludes in inhibiting pancreatic lipase activity, reducing nutrient absorption, impairing adipogenesis, suppressing appetite, enhancing cellular oxidation defenses, activating AMP-activated protein kinase in the skeletal muscle, adipose tissues, and liver, and improving systemic inflammation, hyperglycemia, and insulin resistance [56–59]. Through these diverse mechanisms tea catechins reduce obesity and decrease the adverse health effects of obesity and related complications [59, 61].

Carob (*Ceratonia siliqua*) is a large leguminous tree of the Mediterranean area, belonging to the Caesalpiniaceae family. The plant is used in TAPHM for the treatment of abdominal pain, diarrhea, stomach ulcer, diabetes, chest pain, cough, and urinary tract infections [40, 41]. Carob is utilized mainly for the industrial conversion of the seeds, to obtain locust bean gum or carob gum and germ flour as a by-product, which are used in food preparations as a thickening agent and a source of protein, respectively [64]. The locust bean gum has been shown to possess lipid-lowering effects and to inhibit acylated ghrelin, the hunger hormone. This hormone has effect on making subjects more satiated at meals and making satiation last a long time, decreasing postprandial responses of triglycerides and nonesterified fatty acids, and altering respiratory amount, suggesting a change towards enhanced fatty acid oxidation [65, 66].

In this study the leaves, pods, and seeds of carob were analyzed for their PPLI activity; the leaves and seeds of the plant have shown possessing strong PPLI activity with IC_50_ of 0.8 and 3.3 mg/mL, respectively. This can be attributed to the fact that carob products have been found to contain an array of bioactive compounds including polyphenols and flavonoids that possess antiobesity properties [67]. On the other hand, the seeds of the plant have been shown to contain various bioactive flavonoid compounds including epicatechin, epicatechin gallate, and myricetin with higher concentrations than in pods [67, 68].

A study performed by Vaya & Mahmood [69] has shown that carob leaves contain nine different flavonoids, with myricetin as the major constituent. Interestingly, Su et al. [70] showed that consumption of myricetin may help to prevent obesity and obesity-related metabolic complications. They found myricetin treatment to effectively decrease the size and number of epididymal adipocytes and hepatocytes and reduce fat mass compared with the high-fat diet- (HFD-) induced obesity in mice without myricetin. It was suggested that that myricetin may suppress the expression of adipogenic and lipogenic genes, thereby regulating the onset of obesity [71, 72].

In vivo studies on the effects of polyphenol-rich infusion from carob leaves on inflammation associated with obesity and dextran sulfate sodium- (DSS-) induced ulcerative colitis in Swiss mice showed that carob leaf infusion decreased inflammation severity linked with HFD-induced obesity and dextran sulfate sodium- (DSS-) induced acute colitis designated by reduction in proinflammatory cytokines expression in adipose tissue, colon, and spleen [73]. The anti-inflammatory effect of carob leaves was attributed to its polyphenols which might relieve inflammation severity linked with obesity and colitis.

**Figure 5:**
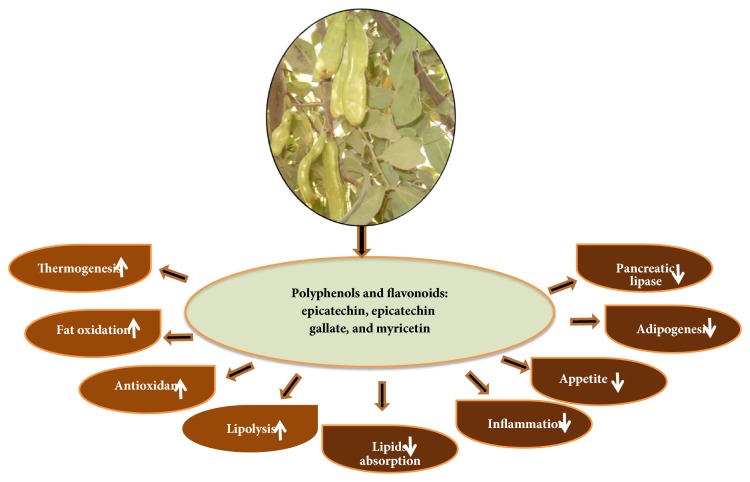
Antiobesity mechanisms and related beneficial effects of* Ceratonia siliqua* extracts and their bioactive constituents.

The present results as well as a review of the available literature have led us to believe that carob products can exert their antiobesity effect through the following main basic mechanisms (Figure 5): inhibiting pancreatic lipase activity, impairing adipogenesis, promoting lipolysis, increasing thermogenesis and fat oxidation, suppressing chronic low-grade inflammation, enhancing cellular antioxidant defenses, reducing absorption of lipids, and controlling appetite. However, the relative role of these mechanisms would depend on the type of carob product and diet consumed by individuals as well as the dietary conditions. Our results indicate that carob products might be a powerful lipase inhibitor and could be used as a weight control in obese patients.

*Curcuma longa* (turmeric) is a rhizomatous herbaceous perennial plant of the ginger family, Zingiberaceae. Turmeric is a commonly used spice and is well documented for its medicinal effects in Indian and Chinese medical systems; the plant is recognized for its anti-inflammatory, antiangiogenic, antioxidant, wound healing, and anticancer properties and health promoting properties [74, 75]. In TAPHM the rhizomes of the plant have been reported to be used for the treatment of several ailments including flatulence, intestinal worms, hepatitis, atherosclerosis, and skin wounds and burns [41].

In the present study, the plant rhizomes exhibited strong PPLI activity (97.4 %, IC_50_ 0.8 mg/mL). This is in agreement with those of Yadav and Chaudhurym [76] and Witkin and Li [77] who also reported a significant antiobesity activity of the plant. Curcumin is the main constituent of the plant; it promotes weight loss and reduces the incidence of obesity-related diseases by inhibiting preadipocyte differentiation, inhibiting macrophage expansion and infiltration in white adipose tissue, suppressing inflammatory adipokine secretion from white adipose tissue, and promoting cytoprotective antioxidant expression [78].

*Mentha spicata* (spearmint) is belonging to the Lamiaceae family. Its extracts (juice and leaf oil) are known to possess several pharmacologic activities, including antioxidant [79], antimicrobial [80], antitumor [81], anti-inflammatory [82], and diuretic [83] properties. It is commonly used in TAPHM in the form of tea as a home remedy to help alleviate stomach pain, to stimulate digestion, notably of fats, and to treat obesity, muscle spasm, headache, menstrual pain, and other digestive system ailments [41]. Mint was among the 39 plants reported in TAPHM to be utilized for weight loss. The PPLI results of the mint extract in this study are in accordance with its traditional use in Palestine and other countries for the treatment of obesity [41].

**Figure 6:**
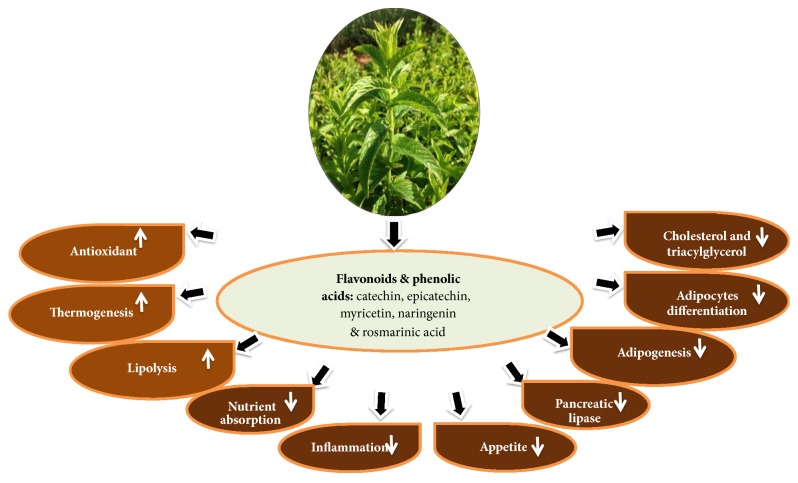
Antiobesity mechanisms and related beneficial effects of* Mentha spicata* extracts and their bioactive constituents.

The* M. spicata *extract contains several bioactive ingredients with known antiobesity properties and is potentially useful in treatment of obesity and overweight related complications (e.g., diabetes and cardiovascular diseases). These compounds include flavonoids such as catechin, epicatechin, rutin, myricetin, luteolin, apigenin, naringenin, kaempferol, and quercetin and phenolic acids such as rosmarinic, gallic, chlorogenic, and caffeic acids [84–88]. Different mechanisms can therefore be proposed to explain the weight-reducing effects of* M. spicata* including inhibiting pancreatic lipase activity, promoting lipolysis, decreasing nutrient absorption, reducing adipocytes differentiation, stimulating thermogenesis and lipid metabolism, inhibiting oxidative stress and inflammation, inhibiting synthesis of plasma, hepatic cholesterol, and triacylglycerol, and controlling appetite (Figure 6).

*Sarcopoterium spinosum* is a widely distributed chamaephyte of the Rosaceae family growing in the eastern Mediterranean region. The shrub is mentioned as a medicinal plant in several ethnobotanical surveys, documenting the use of S. spinosum aqueous root extract by traditional medicinal practitioners for the treatment of diabetes and pain relief (e.g., toothache), as well as cancer therapy [41, 89–92]. The glucose lowering properties of the plant's roots, leaves, and fruits have been demonstrated by Elyasiyan et al. [93]. The plant's glucose lowering agents (bioactive polyphenols, e.g., catechin) target insulin affected tissues (e.g., liver, muscle, and adipose tissue) leading to inhibiting carbohydrate digestion, enhancing insulin secretion, and improving the transmission of insulin signaling in adipocyte and myotypes, leading to enhancing glucose uptake, metabolism, and regulation of whole body energy balance [93, 94].

In our study the plant aerial parts extracts were among the top 5 most active PPLI plant extracts by means of IC_50_ (1.2 mg/mL). This can be attributed to the fact that the* Sarcopoterium* extracts contain an array of bioactive ingredients including polyphenols and flavonoids known as catechins, which include (−)-epigallocatechin gallate, (−)-epicatechin gallate, (−)-epicatechin, (−)-epigallocatechin [92, 95, 96], and triterpenoids such as tormentic acid [97, 98] and *α*-tocopherol [99] that possess antiobesity properties [93]. Our results are in concordance with those of Tovit et al. [100] and Rozenberg et al. [94] which also suggest that the bioactive plant extracts have various beneficial antiobesity effects such as inhibiting lipolysis in an adipocyte, inducing glucose uptake in a cell, reducing plasma glucose levels, preventing atherosclerosis, decreasing weight gain, decreasing food consumption, and/or reducing or preventing obesity.

**Figure 7:**
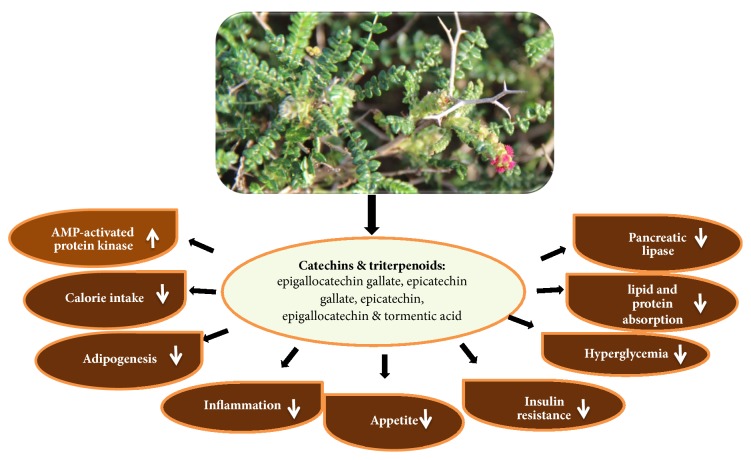
Antiobesity mechanisms and related beneficial effects of* Sarcopoterium spinosum* extracts and their bioactive constituents.

Sarcopoterium-derived polyphenols are expected to reduce obesity and decrease the adverse health effects of obesity and related complications by producing different physiological effects, including weight-reducing effects through the following basic mechanisms (Figure 7): inhibiting pancreatic lipase activity and decreasing absorption of lipids and proteins in the intestine, thus reducing calorie intake, impairing adipogenesis, suppressing appetite, enhancing the activation of AMP-activated protein kinase in the skeletal muscle, adipose tissues, and liver, and improving systemic inflammation, hyperglycemia, and insulin resistance [56, 58, 59, 93].

Table 2 shows that antilipase activity is not necessarily associated with antioxidant activity; however, there were interesting coincidences in the case of* Camellia sinensis*,* Ceratonia siliqua, *and* Curcuma longa*. Eight plant extracts which show strong PPL inhibitory activity have shown possessing weak antioxidant activity by both methods. These include* Elaeagnus angustifolia, Alchemilla xanthochlora*,* Cassia senna*,* Malva sylvestris, Cuminum cyminum*,* Cynara scolymus* (fruit bulb and husk), and* Eruca sativa;* these plants can be considered as potential PPL inhibitors but not as antioxidants. On the other hand, the epicarp of* Rhus coriaria*, the seeds of* Lepidium sativum, *and the leaves of* Cupressus sempervirens *and* Olea europaea *could be considered as alternatives to antioxidants, but not as antilipase sources.

It is worth noting that 76.9 % of the plants analyzed in this study were reported to be used in Palestine as food either in the form of tea, salad, and condiment or in cooked form [51]; of these plants 41.7 % ( 25 plant extracts) possess PPLI activity of more than 50 %. The use of these plants as food suggests that they are safe and can be used in the management of obesity and as natural antioxidants. All plant species analyzed in this study for their antioxidant and PPLI potentials were mentioned to be used in TAPHM for the treatment of several human ailments [40]. On the other hand, of the 41 plant extracts reported by the Palestinians to be used for the treatment of obesity (Table 1), 18 plant extracts have shown to possess PPLI activity of > 50 %. Our results support these ethnobotanical studies and suggest that these plants may serve as the basis for the development of novel antioxidants and antiobesity pharmaceuticals and nutraceuticals/functional foods.

## 5. Conclusions

Our results suggest natural resources that possess strong antioxidant and pancreatic lipase inhibitory activities with potential applications in the treatment and prevention of obesity and overweight problem. The extracts of* Camellia sinensis, Ceratonia siliqua, Curcuma longa*,* Sarcopoterium spinosum*, and* Mentha spicata* have shown possessing strong antioxidants and PPLI potentials. However, future studies are needed for screening in-depth phytochemical, clinical, and possible studies on molecular mechanism of action and identification of the constituents responsible for the antioxidant and PPLI activities. At the same time, efforts should be made to normalize the plant extracts with potent antioxidants and PPLI activities and formulate best alternative herbal products in order to substitute manmade drugs which are presently in use.

## Figures and Tables

**Table 1:** Plants used in Traditional Arabic Palestinian Herbal Medicine for the treatment of obesity.

Plant Derived Product (Family)	English Common Name	Plant Part	Mode of Preparation
*Alchemilla xanthochlora* Rothm. (Alchemilla *vulgaris* auct.) (Rosaceae)	Lion's Foot, Lady's – Mantle	AP	(i) A decoction is prepared from the plant; take 1 cup twice daily. (ii) A decoction is prepared from plant mixed with rosemary, and wild thyme. 1-2 cups are taken daily

*Allium cepa *L. (Liliaceae)	Onion	FLE	(i) Add 1 teaspoon of the plant juice to a cup of fruit juice to sweeten the taste, and drink 1 cup daily

*Allium sativum *L. (Liliaceae)	Garlic	GLV	(i) Eaten raw before breakfast

*Brassica oleracea *L. (Brassicaceae)	Wild Cabbage	AP	(i) Eaten raw as salad, or cooked as vegetables. (ii) A decoction is prepared from the plant leaves. 1 cup is taken daily for 4-5 months

*Brassica oleracea* var. *botrytis* L. (Brassicaceae)	Cauliflower	FR	(i) Eaten raw as salad

Camellia sinensis (L.) Kuntze (Theaceae)	Tea	LE	(i) A standard decoction is prepared from the plant. Take 2 cups daily

*Cassia senna *L. (Fabaceae)	Senna	LE, SD	(i) Mix equal amounts of the leaves of senna, cumin, fennel, and anise leaves; eat one teaspoon daily after lunch. (ii) A decoction is prepared from the leaves or the seeds; 1 cup is taken daily.

*Cichorium pumilum *Jacq. (Asteraceae)	Dwarf Chicory	AP	(i) A decoction is prepared from the leaves. Take 3 cups daily. (ii) Smash 5 teaspoons of the roots, mix with 1 L of water, and boil for 15 minutes. 1 cup is taken before meals

Cinnamomum verum J. Presl (Lauraceae)	Cinnamon Tree	BRK	(i) A standard decoction is prepared from the plant. Take 2 cups daily

*Citrullus lanatus *(Thunb.) Matsum & Nakai (Cucurbitaceae)	Watermelon	FR	(i) Eaten raw between meals

*Citrus limon *(L.) Burm. Fil (Rutaceae)	Lime, Limon Tree	FR	(i) A juice is prepared from the fruit; one teaspoon is taken daily before breakfast (ii) A standard decoction is prepared from the leaves. 1 cup is taken daily before breakfast.

*Citrus paradisi *Macfad. (Rutaceae)	Grapefruit	FR	(i) Fruits are eaten raw or drunk as a juice 1- 2 cups daily before breakfast

*Crataegus aronia *(L.) Bosc. ex DC. (Rosaceae)	Hawthorn	LE	(i) A decoction is prepared from the leaves. 1-3 cups are taken daily

*Cucumis sativus *L. (Cucurbitaceae)	Cucumber	FR	(i) Eaten raw between meal

*Cuminum cyminum* L. (Apiaceae)	Cumin	SD	(i) Mix equal amounts of the seeds of cumin, fennel, anise, and senna leaves; eat one teaspoon daily after lunch. (ii) Soak the ground seeds overnight in boiled water and lemon. Drink the filtrate in the morning before breakfast for a month

*Cynara scolymus *L. (Asteraceae)	Artichoke	FR	(i) Fruits are eaten raw as salad

*Eriobotrya japonica *L. (Rosaceae)	Medlar Tree	LE	(i) A decoction is prepared from the plant leaves and olive leaves. Drink 1 cup twice a day.

*Eruca sativa *Miller (Brassicaceae)	Garden Rocket	AP	(i) Eaten raw with salad

*Foeniculum vulgare *Miller (Apiaceae)	Fennel	LE	(i) Leaves are eaten with salad. (ii) Seeds are boiled in water for 20 minutes; drink 2 cups daily for 3-4 weeks

*Lactuca sativa *L. (Asteraceae )	Lettuce	AP	(i) Eaten fresh with salad.

*Lupinus albus* L. (Fabaceae)	Lupine	SD	(i) Seeds are soaked in water for few days and then eaten raw. (ii) 1 teaspoon of the grinded seeds is taken daily. (iii) The ground seeds are mixed with fenugreek seeds and honey; 1 teaspoon is taken daily.

*Malva sylvestris *L. (Malvaceae)	Common Mallow	LE	(i) Cooked as vegetables

*Matricaria aurea *(L.) Sch. Bip. (Asteraceae )	Golden Cotula	AP	(i) Leave are soaked in hot water for 2 hours. Drink 1 cup twice daily. (ii) Add 3 teaspoons of the flowers to boiling water, filtrate, and have 1 cup twice a day for 1 month

*Mentha spicata *L. (Lamiaceae)	Peppermint	AP	(i) A decoction is prepared from the plant. 1 cup is taken when needed

*Olea europaea *L. (Oleaceae)	Olive	LE	(i) A decoction is prepared from the plant leaves and meddler tree leaves. Drink 1 cup twice a day.

*Origanum syriacum *L. (Lamiaceae)	Wild Thyme, Mother of Thyme	AP	(i) A decoction is prepared from the plant; take 1 cup twice daily. (ii) A decoction is prepared from plant mixed with Lion's Foot, rosemary, and wild thyme. 1-2 cups are taken daily

*Paronychia argentea *Lam. (Caryophyllaceae)	Silvery Whitlow- Wart	AP	(i) A decoction is prepared from plant mixed with lion's foot, rosemary, and wild thyme. 1-2 cups are taken daily

*Petroselinum sativum *Hoffm. (Apiaceae)	Parsley	AP	(i) A standard decoction is prepared from the plant; take 1-3 cups/day. (ii) Eaten raw with salad

*Pimpinella anisum *L. (Apiaceae)	Anise	SD	(i) Mix equal amounts of the seeds of anise, cumin, fennel, and senna; eat one teaspoon daily after lunch.

*Pistacia palaestina *Boiss. (Anacardiaceae)	Palestinian pistachio	LE	(i) A standard decoction is prepared from the plant; take 2 cups three time a day

*Portulaca oleracea* L. (Portulacaceae)	Purslane	SD	(i) A standard decoction is prepared from the seeds; take 1-2 cups daily

*Punica granatum *L. (Punicaceae)	Pomegranate	HS	(i) The husk is dried, ground, and then soaked in water. 1 cup is taken daily.

*Pyrus malus * L. (Rosaceae)	Apple	FR	(i) Add one teaspoon of apple vinegar to a glass of water; drink 3 times daily after meals. (ii) Drink 1 teaspoon of apple vinegar before breakfast.

*Rosmarinus officinalis* L. (Lamiaceae)	Rosemary	AP	(i) A decoction is prepared from the plant; take 1 cup twice daily. (ii) A decoction is prepared from plant mixed with Lion's Foot, Silvery Whitlow-Wart, and wild thyme. 1-2 cups are taken daily

*Salvia fruticosa* Mill. (Lamiaceae)	Common Sage	LE	(i) A standard decoction is prepared from the plant. Take 2-3 cups daily for a month.

*Teucrium capitatum* L. (*T. polium* L.) (Lamiaceae)	Cat Thyme	LE	(i) A standard decoction is prepared from the plant; take 1 cup/day when needed.

*Trigonella berythea *Boiss. & Blanche(*T. foenum- graecum *L.) (Fabaceae)	Fenugreek	SD	(i) A decoction is prepared from 1 teaspoon of the seeds in 500 ml of water; take 1-2 cups daily. (ii) The ground seeds are mixed with fenugreek seeds and honey; 1 teaspoon is taken daily.

*Varthemia iphionoides *Boiss & Blanche (*Chiliadenus iphionoides*)(Asteraceae)	Common Varthemia	AP	(i) A standard decoction is prepared from the plant. 1 cup is taken twice a day

*Zingiber officinale *Rose. (Zingiberaceae)	Ginger	Rz	(i) A decoction is prepared from the rhizomes. 1 cup is taken daily

*∗*RZ: rhizome; HS: husk; AP: aerial parts; LE: leave; FR: fruits; SD: seeds; BRK: bark; FLE: fleshy leaves; GLV: gloves.

**Table 2:** Antioxidant and pancreatic lipase inhibition activity of Palestinian plants used in Traditional Arabic Palestinian Herbal Medicine (TAPHM).

Scientific name (Family)	Common name	Plant part	Arabic name	Antioxidant capacity					Lipase inhibition	
				DPPH			Reducing Power			
				% Inhibition (1mg/ml)	IC50 (mg/ml)	AAI	ABS-700 nm	EC50 (mg/ml)	% Inhibition (5mg/ml)	IC50 (mg/ml)
*Camellia sinensis* (L) Kuntze (Theaceae)	Tea	LE	Shai	86.2±4.1	0.10±0.00	1±0.02	2.26±0.1	0.11±0.0	99.0±1.5	0.45±0.2
*Ceratonia siliqua* L. (Caesalpiniaceae)	Carob	LE	Kharrob	91.2±3.1	0.04±0.00	2.63±0.1	2.6±0.1	0.05±0.1	95.4±8.0	0.76±0.4
*Curcuma longa *L. (Zingiberaceae)	Turmeric	RZ	Kurkum	79.5±0.9	0.39±0.10	0.26±0.05	0.87±0.03	0.59±0.1	97.4±0.8	0.82±0.3
*Sarcopoterium spinosum* (L.) Spach. (Rosaceae)	Shrubby Barnet	AP	Natesh	68.2±7.7	0.02±0.01	4.08±0.9	2.56±0.03	0.04±0.0	95.3±15.0	1.17±0.2
*Mentha spicata* L. (Lamiaceae)	Peppermint	AP	Na'na'	74.0±0.2	0.21±0.03	0.47±0.05	2.04±0.03	0.15±0.02	92.5±2.0	1.19±1.0
*Rhus coriaria* L. (Anacardiaceae)	Sicilian Sumac	LE	Summaq	95.1±3.	0.02±0.01	4.67±0.1	2.61±0.03	0.005±0.0	95.1±7.6	1.24±0.2
*Dittrichia viscosa* (L.) Greuter (Asteraceae)	Inula	AP	Irq tayyon	86.5±0.	0.11±0.01	0.94±0.1	2.33±0.1	0.13±0.01	78.5±14.8	1.28±0.3
*Rosmarinus officinalis* L. (Lamiaceae)	Rosemary	AP	Hasalban	89.9±0.5	0.09±0.02	1.11±0.3	2.28±0.08	0.16±0.01	94.9±4.1	1.31±0.6
*Eruca sativa* Miller (Brassicaceae)	Garden Rocket	AP	Jarjeer	21.7±0.5	NA*∗∗*	NA	0.8±0.03	NA	74.9±2.6	1.45±0.3
*Elaeagnus angustifolia* L. (Elaeagnaceae)	Narrow-Leaved Oleaster	LE	Zaizafon	51.9±1.8	NA	NA	0.46±0.1	NA	96.8±2.2	1.51±0.2
*Teucrium capitatum* L. (*T. polium* L.) (Lamiaceae)	Cat Thyme	AP	Je'det Alsobian	85.3±6.1	0.12±0.01	0.82±0.1	1.66±0.02	0.41±0.01	79.2±14.5	1.65±0.5
*Coridothymus capitatus* (L.) Reichb. (Lamiaceae)	Capitate Thyme	LE	Za'tar Farsi	88.0±0.2	0.07±0.01	1.39±0.2	2.43±0.02	0.14±0.01	78.1±22.2	1.92±0.1
*Malva sylvestris* L. (Malvaceae)	Common Mallow	AP	Khubeizeh	21.3±1.2	NA	NA	0.37±0.01	NA	72.7±5.6	1.97±0.9
*Ziziphus sativa* Gaertn. (Rhamnaceae)	Jujube	LE	Inab	84.9±0.2	0.10±0.01	0.96±0.1	1.87±0.4	0.18±0.1	84.0±8.1	1.99±0.8
*Punica granatum* L. (Punicaceae)	Pomegranate	HS	Rumman	78.7±3.30	0.05±0.01	2.04±0.4	2.62±0.01	0.03±0.0	92.5±5.8	2±0.2
*Cassia senna* L. (Fabaceae)	Senna	LE	Sanamaki	31.2±0.12	NA	NA	0.47±0.02	NA	81.8±12.8	2.32±0.5
*Citrus limon* (L.) Burm. Fil (Rutaceae)	Lime, Limon Tree	LE	Laimoun	84.7±2.10	0.43±0.10	0.23±0.03	0.52±0.06	1.19±0.02	82.2±10.8	2.46±0.2
*Cynara scolymus* L. (Asteraceae)	Artichoke	FR	Kharshouf	3.8±0.51	NA	NA	0.13±0.01	NA	73.5±1.7	2.68±0.2
Crataegus aronia (L.) Bosc. ex DC. (Rosaceae)	Hawthorn	LE	Za'rour Sha'ek	85.0±0.31	0.51±0.06	0.2±0.1	0.73±0.03	0.71±0.02	72.7±8.9	2.73±0.04
*Portulaca oleracea* L. (Portulacaceae)	Purslane	SD	Baqleh	88.9±0.71	0.41±0.08	0.25±0.03	1.28±0.01	0.33±0.01	72.2±4.8	2.77±0.6
*Juglans regia* L. (Juglandaceae)	Walnut	LE	Jouz	82.2±3.22	0.14±0.04	0.71±0.2	1.68±0.03	0.39±0.03	69.1±8.3	2.81±0.2
*Arum palaestinum* Boiss. (Araceae)	Palestinian Arum	LE	Lufe	83.8±0.58	0.49±0.03	0.2±0.0	0.67±0.01	0.77±0.00	76.5±9.2	2.88±0.8
*Quercus calliprinos* Webb (Fagaceae)	Kermes Oak	LE	Sendian	76.1±3.20	0.08±0.06	1.23±1.5	2.66±0.01	0.05±0	71.2±9.7	2.88±0.1
*Cichorium pumilum *Jacq. (Asteraceae)	Dwarf Chicory	LE	Hindba'	82.7±1.60	0.6±0.07	0.17±0.03	0.79±0.01	0.7±0.02	71.3±10.2	2.94±0.8
*Foeniculum vulgare *Miller (Apiaceae )	Fennel	LE	Shoumer	74.9±0.7	0.37±0.03	0.27±0.1	0.74±0.02	0.69±0.01	83.2±8.5	3.01±0.2
*Cuminum cyminum* L. (Apiaceae )	Cumin	SD	Kamoon	45.0±0.5	NA	NA	0.46±0.01	NA	72.9±5.2	3.01±0.0
*Ziziphus spina-christi* (L.) Desf. (Rhamnaceae)	Christ's Thorn Jujube	LE	Seder	86.2±2.6	0.12±0.02	0.82±0.2	1.51±0.13	0.4±0.02	70.1±6.9	3.11±0.1
*Cynara scolymus* L. (Asteraceae)	Artichoke	FR	Kharshouf	8.1±0.2	NA	NA	0.17±0.03	NA	75.7±2.4	3.16±0.5
*Portulaca oleracea* L. (Portulacaceae)	Purslane	AP	Baqleh	82.9±0.9	0.53±0.07	0.19±0.01	1.21±0.02	0.46±0.00	72.9±11.8	3.21±0.3
*Satureja thymbra* L. (Lamiaceae)	Summer Savory	AP	Za'tar	82.9±2.8	0.34±0.29	0.29±0.4	1.93±0.09	0.18±0.01	60.9±9.5	3.31±0.1
Ceratonia siliqua L. (Caesalpiniaceae)	Carob	SD	Kharrob	89.0±1.4	0.60±0.02	0.17±0.01	1.42±0.11	0.35±0.03	68.7±6.2	3.3±0.4
*Alchemilla xanthochlora* Rothm. (*Alchemilla vulgaris* auct.) (Rosaceae)	Lion's Foot, Lady's – Mantle	LE	Rijl Alasad, Kaf Alasad	19.0±0.23	NA	NA	0.43±0.01	Na	77.4±19.1	3.36±0.1
*Salvia fruticosa* Mill. (Lamiaceae)	white sage, Common Sage, Garden Sage	AP	Maryemeyeh	91.0±0.6	0.13±0.00	0.76±0.02	1.51±0	0.32±0.1	65.8±2.1	3.45±0.4
*Psidium guajava* L. (Myrtaceae)	Guava	LE	Guava	69.6±0.2	0.09±0.01	1.11±0.1	2.29±0.02	0.12±0.02	55.4±2.8	3.47±1.6
*Trigonella berythea* Boiss. & Blanche(*T. foenum- graecum *L.) (Fabaceae)	Fenugreek Seed	SD	Hilbeh	60.2±7.6	1.02±0.04	0.1±0.0	0.22±0.01		63.8±5.3	3.67±0.1
*Paronychia argentea *Lam. (Caryophyllaceae)	Silvery Whitlow- Wart	AP	Rijl Alhamameh	78.7±0.7	0.38±0.02	0.26±0.1	1.05±0.01	0.43±0.01	68.4±17.1	4.23±1.1
*Ficus carica* L. (Moraceae)	Fig Tree	LE	Teen	82.2±0.3	0.29±0.00	0.34±0.0	0.71±0.02	0.88±0.09	49.5±7.9	NA
*Capparis spinosa* L. (Capparaceae)	Caper Bush, Egyptian Caper	LE	Kappar	90.47±4.0	0.24±0.15	0.42±0.3	1.09±0.02	0.52±0.07	49.0±7.7	NA
*Solanum nigrum *L. (Solanaceae)	Nightshade	AP	Samweh	83.0±4.0	0.44±0.02	0.23±0.01	0.78±0.13	0.8±0	46.3±4.5	NA
*Origanum majorana* L. (Lamiaceae)	Sweet-Marjoram	AP	Mardaqoush	82.1±0.1	0.23±0.12	0.44±0.3	2.06±0.02	0.26±0.02	46.1±5.6	NA
*Euphorbia hierosolymitana* Boiss. (Euphorbiaceae)	Spurge	AP	Halabloub	90.5±4.7	0.33±0.08	0.3±0.1	0.85±0.02	0.59±0.02	45.8±2.8	NA
*Eucalyptus camaldulensis* Dehn. (Myrtaceae)	Red River Gum	LE	Kina	87.9±4	0.09±0.03	1.11±0.4	2.37±0.02	0.15±0.01	44.3±5.4	NA
*Origanum syriacum* L. (Lamiaceae)	Wild Thyme, Mother of Thyme	AP	Za'tar bari	81.4±1.8	0.3±0.08	0.33±0.1	1.02±0.03	0.57±0.02	41.5±1.6	NA
*Cinnamomum zeylanicum* Blume. (Lauraceae)	Cinnamon Tree	BRK	Qerfeh	86.4±1.9	0.06±0.02	1.54±0.4	2±0.18	0.24±0.02	40.5±8.6	NA
*Matricaria aurea* (L.) Sch. Bip. (Asteraceae)	Golden Cotula	AP	Babounej	75.1±14.6	0.39±0.06	0.26±0.04	0.82±0.02	0.69±0.09	38.5±2.7	NA
*Nigella ciliari*s DC. (Ranunculaceae)	Nigella, Black Cumin	SD	Qezha	34.4±9.0	NA	NA	0.19±0.03	NA	38.4±3.2	NA
*Syzygium aromaticum* (L.) Merr. and Perry. (Myrtaceae)	Clove	FLW	Kabsh Koronful	80.2±2.3	0.15±0.01	0.65±0.04	2.46±0.06	0.09±0.01	37.1±0.9	NA
*Olea europaea* L. (Oleaceae)	Olive	LE	Zaitoun	91.1±2.5	0.29±0.02	0.35±0.02	1.02±0.05	0.54±0.04	36.8±1.3	NA
*Pimpinella anisum *L. (Apiaceae )	Anise	SD	Yansoon	83.3±4.0	0.47±0.01	0.21±0.0	0.63±0.04	1.06±0.12	35.4±2.0	NA
*Pinus halepensis* Mill. (Pinaceae)	Aleppo Pine	AP	Sonawabar halabi	46.5±7.7	NA	NA	0.48±0.06	NA	32.5±6.1	NA
*Passiflora incarnata* L. (Passifloraceae)	Passion flower	LE	Pasiflora	79.2±6.0	0.74±0.04	0.13±0.01	0.53±0.24	1.6±0.61	32.3±2.8	NA
*Myrtus communis* L. (Myrtaceae)	Common Myrtle	LE	Habaq	81.1±**0.5**	0.17±0.01	0.59±0.02	1.92±0.24	0.25±0.02	30.2±0.1	NA
*Pistacia palaestina* Boiss. (Anacardiaceae)	Palestinian pistachio, Terebinth	LE	Botom Falastini	89.98±1.1	0.17±0.02	0.57±0.1	1.37±0.12	0.32±0	29.0±0.2	NA
*Retama raetam* (Forssk.) Webb (Fabaceae)	Retama, White Broom	AP	Retem	70.4±4.9	0.4±0.06	0.23±0.03	0.48±0.16	NA	28.6±3.9	NA
*Rhus coriaria* L. (Anacardiaceae)	Sicilian Sumac	EPC	Summaq	92.8±1.3	0.02±0.01	4.37±1.32	2.66±0.06	0.06±0	25.5±3.2	NA
*Rhus coriaria* L. (*Anacardiaceae*)	Sicilian Sumac	SD	Summaq	30.2±2.2	NA	NA	0.46±0.18	NA	23.4±2.3	NA
*Lepidium sativum* L.	Cress	SD	Rashad	92.2±0.7	0.60±0.08	0.18±0.03	0.65±0.21	0.94±0.06	22.9±2.6	NA
*Laurus nobilis* L. (Lauraceae)	Laurel, Sweet Bay	LE	Ghar	85.9±0.5	0.50±0.01	0.2±0.0	0.73±0.05	0.82±0.07	22.5±2.6	NA
*Varthemia iphionoides* Boiss & Blanche (*Chiliadenus iphionoides*)* (Asteraceae)*	Common Varthemia	AP	Kteileh	84.6±0.9	0.40±0.01	0.2±0.0	1.12±0.01	0.4±0.03	20.6±5.4	NA
*Capparis spinosa* L. (Capparaceae)	Caper Bush, Egyptian Caper	FR	Kappar	20.1±0.2	NA	NA	0.43±0.13	NA	19.9±19.6	NA
*Lupinus albus* L.(Fabaceae)	Lupine	SD	Tormous Mor	16.2±4.1	NA	NA	0.06±0.01	NA	19.9±6.8	NA
*Ceratonia siliqua L. (Caesalpiniaceae*)	Carob	FR	Kharrob	27.0±0.6	NA	NA	0.28±0.17	NA	17.5±2.5	NA
*Ruta chalepensis* L. (Rutaceae)	Rue	AP	Figan	73.8±0.4	0.34±0.24	0.29±0.28	0.35±0.1	NA	17.1±2.5	NA
*Zingiber officinale* Rose. (Zingiberaceae)	Ginger	RZ	Zanjabeel	79.0±7.8	0.77±0.00	0.13±0.0	0.45±0.01	NA	16.7±7.6	NA
*Foeniculum vulgare* Miller (Apiaceae )	Fennel	SD	Shoumer	87.0±0.6	0.45±0.00	0.22±0.0	0.75±0.22	0.87±0.08	16.5±1.2	NA
*Morus nigra* L. (Moraceae)	Mulberry	FR	Toot	69.4±14.3	1.47±0.35	0.1±0.02	0.18±0.01	NA	16.1±2.3	NA
Elaeagnus angustifolia L. (Elaeagnaceae)	Narrow-Leaved Oleaster	FR	Zaizafon	8.6±0.8	NA	NA	0.17±0.01	NA	15.7±2.4	NA
*Cupressus sempervirens* L. (Cupressaceae)	Cypress	LE	Saro	91.48±1.2	0.26±0.02	0.4±0.03	1.5±0.02	0.33±0.01	15.2±1.0	NA
*Thuja occidentalis* L. (Cupressaceae)	Tree Of Life	LE	Afs	82.0±7.1	0.33±0.11	0.3±0.1	0.75±0.23	0.58±0.02	14.3±11.5	NA
*Phaseolus vulgaris* L. (Fabaceae)	Common Bean	SD	Fasolia'	25.0±2.7	NA	NA	0.32±0.04	NA	13.7±1.1	NA
*Cucumis sativus* L. (Cucurbitaceae)	Cucumber	FR	Khiar	1.0±0.0	NA	NA	0.07±0.00	NA	13.5±2.0	NA
*Punica granatum* L. (Punicaceae)	Pomegranate	SD	Rumman	42.9±0.2	NA	NA	0.19±0.01	NA	12.4±2.7	NA
*Petroselinum sativum* Hoffm. (Apiaceae )	Parsley	AP	Baqdounes	27.7±0.3	NA	NA	0.12±0.01	NA	12.4±0.7	NA
*Citrullus lanatus* (Thunb.) Matsum & Nakai (Cucurbitaceae)	Watermelon	FR	Bateekh	2.8±0.32	NA	NA	0.04±0.00	NA	11.6±0.5	NA
*Brassica olerac*ea var. *botrytis* L. (Brassicaceae)	Cauliflower	FR	Qernabeet	2.0±0.8	NA	NA	0.22±0.05	NA	11.2±1.1	NA
*Brassica oleracea var. botrytis *L. (Brassicaceae)	Cauliflower	FR	Qernabeet	2.0±0.8	NA	NA	0.22±0.05	NA	11.2±1.1	NA
*Aloe vera *(L.) Burm. f. (Xanthorrhoeaceae)	Aloe	LE	Sabrah murrah	31.9±12.0	NA	NA	0.18±0.03	NA	10.1±0.9	NA
*Rubus sanctus *Schreb. (Rosaceae)	Blackberry	LE	Oliq	88.1±4.6	0.4±0.16	0.24±0.1	0.49±0.01	NA	10.1±2.8	NA
*Cichorium pumilum *Jacq. (Asteraceae)	Dwarf Chicory	RT	Hindba'	4.9±0.2	NA	NA	0.13±0.01	NA	10.1±0..0	NA
*Coriandrum sativum *L. (Apiaceae )	Coriander	SD	Kozbareh	85.9±5.6	0.6±0.02	0.16±0.01	0.42±0.16	NA	9.7±2.2	NA
*Sesamum indicum *L. (Pedaliaceae)	Sesame	SD	Semsem	11.0±3.2	NA	NA	0.11±0	NA	9.5±7.4	NA
*Lactuca sativa *L. (Asteraceae)	Lettuce	AP	Khass	28.6±6	NA	NA	0.18±0.04	NA	7.4±0.4	NA
*Brassica oleracea *L. (Brassicaceae)	Wild Cabbage	AP	Malfouf	40.0±10.9	NA	NA	0.27±0.06	NA	6.3±5.4	NA
*Hibiscus sabdariffa *L. (Malvaceae)	Roselle	FLW	Karkadaih	89.1±5.1	0.5±0.05	0.19±0.02	0.62±0.07	0.98±0.1	6.3±0.4	NA
*Citrus paradisi *Macfad. (Rutaceae)	Grapefruit	FR	Laimoon Aljeneh	2.5±0.3	NA	NA	0.1±0.01	NA	4.4±0.2	NA
*Allium cepa *L. (Amaryllidaceae)	Onions	FLE	Basal	18.0±8.1	NA	NA	0.1±0.02	NA	2.5±1.6	NA
*Citrus limon *(L.) Burm. Fil (Rutaceae)	Limon Tree	FR	Laimoun	52.5±0.9	3.6±1.9	0.03±0.02	0.4±0.06	NA	2.3±1.2	NA
*Vitis vinifera *L. (Vitaceae)	Grape	FR	Inab	87.5±0.1	0.5±0.0	0.2±0.00	1.1±0.08	0.34±0.1	2.0±1.6	NA
*Allium sativum *L. (Amaryllidaceae)	Garlic	GLV	Thoum	3.1±5.2	NA	NA	0.1±0.04	NA	2.0±0.2	NA
*Pyrus malus *L. (Rosaceae)	Apple	FR	Tuffah	26.1±7.6	NA	NA	0.1±0.07	NA	1.7±2.4	NA
Orlistat (200 ug/ml)								92±0.2	0.1±0.0	
Ascorbic acid (100ug/ml)			89.2±0	0.03±0.0	3.9±0.01	1.5±0.08	0.06±0.02			
BHT (100ug/ml)			93.4±0.57	0.03±0.0	3.2±0.51	0.8±0.05	0.06±0.00			

*∗*RZ: rhizome; HS: husk; AP: aerial parts; LE: leave; FR: fruits; SD: seeds; BRK: bark; FLW: flowers; EPC: epicarp; RT: roots; FLE: fleshy leaves; GLV: gloves. *∗∗*NA: not applicable.

## Data Availability

The data sets supporting the results of this article will be freely available upon request to the corresponding author (msshtayeh@yahoo.com) for noncommercial use only.

## Abbreviations

AAI: Antioxidant activity index
AC: Absorbance of the negative control
AS: Absorbance of the sample
BMI: Body mass index
DPPH: *α*, *α*-Diphenyl-*β*-picrylhydrazyl
DSS: Dextran sulfate sodium
EC50: The effective concentration at which the absorbance was 0.5
ECG: L-Epicatechin, epicatechin gallate
EGC: Epigallocatechin
EGCG: Epigallocatechin gallate
HFD: High-fat diet
pNPB: p-Nitrophenol butyrate
PPL: Porcine pancreatic lipase
RP: Reducing power
TAPHM: Traditional Arabic Palestinian Herbal Medicine.
